# Cardiac cell proliferation is not necessary for exercise‐induced cardiac growth but required for its protection against ischaemia/reperfusion injury

**DOI:** 10.1111/jcmm.13078

**Published:** 2017-03-17

**Authors:** Yihua Bei, Siyi Fu, Xiangming Chen, Mei Chen, Qiulian Zhou, Pujiao Yu, Jianhua Yao, Hongbao Wang, Lin Che, Jiahong Xu, Junjie Xiao

**Affiliations:** ^1^ Cardiac Regeneration and Ageing Lab School of Life Science Shanghai University Shanghai China; ^2^ Department of Clinical laboratory Nanxiang Hospital of Jiading Shanghai China; ^3^ Department of Geriatrics Xuhui Central Hospital Shanghai Clinical Center Chinese Academy of Science Shanghai China; ^4^ Department of Cardiology Tongji Hospital Tongji University School of Medicine Shanghai China; ^5^ Department of Cardiology Shanghai Yangpu District Hospital Tongji University School of Medicine Shanghai China

**Keywords:** exercise, proliferation, 5‐fluorouracil, ischaemia/reperfusion injury

## Abstract

The adult heart retains a limited ability to regenerate in response to injury. Although exercise can reduce cardiac ischaemia/reperfusion (I/R) injury, the relative contribution of cardiac cell proliferation including newly formed cardiomyocytes remains unclear. A 4‐week swimming murine model was utilized to induce cardiac physiological growth. Simultaneously, the antineoplastic agent 5‐fluorouracil (5‐FU), which acts during the S phase of the cell cycle, was given to mice *via* intraperitoneal injections. Using EdU and Ki‐67 immunolabelling, we showed that exercise‐induced cardiac cell proliferation was blunted by 5‐FU. In addition, the growth of heart in size and weight upon exercise was unaltered, probably due to the fact that exercise‐induced cardiomyocyte hypertrophy was not influenced by 5‐FU as demonstrated by wheat germ agglutinin staining. Meanwhile, the markers for pathological hypertrophy, including ANP and BNP, were not changed by either exercise or 5‐FU, indicating that physiological growth still developed in the presence of 5‐FU. Furthermore, we showed that CITED4, a key regulator for cardiomyocyte proliferation, was blocked by 5‐FU. Meanwhile, C/EBPβ, a transcription factor responsible for both cellular proliferation and hypertrophy, was not altered by treatment with 5‐FU. Importantly, the effects of exercise in reducing cardiac I/R injury could be abolished when cardiac cell proliferation was attenuated in mice treated with 5‐FU. In conclusion, cardiac cell proliferation is not necessary for exercise‐induced cardiac physiological growth, but it is required for exercise‐associated protection against I/R injury.

## Introduction

Exercise is now accepted as a useful therapeutic strategy for cardiovascular diseases 1. Although similar in morphological level to pathological hypertrophy, exercise‐induced cardiac growth has distinct cellular and molecular mechanisms 2. Substantially, the adult mammalian heart has some endogenous regenerative capacity which could be enhanced by exercise 3. However, the contribution of cardiomyocyte renewal to the benefit of exercise upon cardiac injury is largely unknown 4.

Various studies have reported that exercise can attenuate cardiac I/R injury 5, 6. The cardiac cell death due to apoptosis and necrosis represents key cellular responses upon myocardial I/R injury 7. Exercise was demonstrated to reduce cardiomyocyte apoptosis in a PI3 kinase‐ and Akt‐dependent manner 8. The anti‐apoptosis effect on cardiomyocytes is also linked to enhanced antioxidative defence and mitochondrial adaptions upon exercise 9, 10, 11. Meanwhile, exercise is able to improve myocardial glucose and fatty acid metabolism during I/R injury 12. Noteworthy, exercise‐induced cardiomyocyte renewal is supposed to be able to partly make up the cell loss during cardiac I/R injury, although the relative contribution has not yet been elucidated 13.

In this study, we utilized a mouse swimming exercise model to induce cardiac physiological growth. Simultaneously, the antineoplastic agent 5‐FU, which acts during the S phase of the cell cycle by inhibiting DNA synthesis and reducing cell proliferation, was given to mice *via* intraperitoneal injections. This study aimed firstly to clarify whether cardiac cell proliferation is necessary for exercise‐induced cardiac growth at both cellular and molecular levels, and then to elucidate whether or to what extent cardiac cell proliferation contributes to the protective effect of exercise against cardiac I/R injury.

## Materials and methods

### Animals

Male C57BL/6 mice aged 8 weeks were purchased from the Animal Research Center of Fudan University (Shanghai, China) and maintained in SPF laboratory Animal Facility of Shanghai University (Shanghai, China). All animal experiments were conducted under the guidelines on the use and care of laboratory animals for biomedical research published by National Institutes of Health (No. 85‐23, revised 1996). This study was approved by the committee on the Ethics of Animal Experiments of Shanghai University.

### Mouse swimming protocol

Adult male C57BL/6 mice were submitted to a 4‐week swimming exercise to induce cardiac physiological growth 13. Briefly, mice received a swimming protocol which started from 5 min. twice for the first day and 10 min. twice for the second day, and then continued with an increase of 10 min. per day until 90 min. twice per day reached. After 4 weeks of exercise training, mice were killed and tissues were collected. The heart weight (HW), heart weight/body weight ratio (HW/BW) and heart weight/tibia length ratio (HW/TL) were calculated to evaluate cardiac growth after exercise.

### Cardiac I/R model

By the day of the last swimming session, cardiac I/R injury was induced by 30 min. of coronary artery ligation followed by 24 hrs of cardiac reperfusion as previously described 14. Briefly, the left anterior descending artery (LAD) was ligated with 8–0 silk in anesthetized mouse. After 30 min. of LAD occlusion, the ligature was relieved. Twenty‐four hours after reperfusion, mice were killed and hearts were stained with 2,3,5‐triphenyltetrazolium chloride (TTC) as reported previously 15. In brief, mice were anesthetized with an intraperitoneal injection of 0.5 mg/g of tribromoethanol. After 1 ml of Evans blue (Biosharp, Anhui, China) was slowly injected into the inferior vena, the heart was quickly removed and stored at −20°C for 15 min. Then, the heart was cut into 5 transverse slices at 1 mm thickness across the long axis and stained with 1% TTC in phosphate‐buffered saline (PBS) at 37°C for 10 min. After fixed with 4% paraformaldehyde (PFA), images were taken under light microscope and analysed with IMAGEJ software (National Institutes of Health, Bethesda, MD, USA). The area at risk/left ventricle weight (AAR/LV) ratio was calculated to evaluate the stability and homogeneity of surgery. The infarct area/area at risk (INF/AAR) ratio was calculated to delineate the degree of cardiac I/R injury.

### 5‐FU and EdU injections

As we do not have a method to specifically inhibit cardiomyocyte proliferation, we investigated whether cardiac cell proliferation was necessary for exercise‐induced cardiac growth and to what extent it contributed to the cardiac protective effect of exercise. 5‐FU injection was performed in the present study to block cell proliferation. Briefly, mice were intraperitoneally injected with 5‐FU (10 mg/kg; Sigma‐Aldrich, St. Louis, MO, USA) every 5 days for five cycles, starting from the third day of swimming training 16. The control mice were injected with PBS instead. For mice enrolled for EdU incorporation assay, 5‐ethynyl‐2′‐deoxyuridine (EdU, 50 mg/kg; Invitrogen, Carlsbad, CA, USA) was intraperitoneally injected to mice the day before killing.

### EdU and Ki‐67 immunofluorescent staining

The 5‐μm‐thick frozen sections of heart tissues were fixed with 4% PFA for 15 min. and washed with PBS for three times. Next, the heart sections were treated with 0.5% Triton X‐100 for 20 min., and then blocked in 3% bovine serum albumin (BSA) for 1 hr at room temperature. Afterwards, the sections were incubated with rabbit polyclonal Ki‐67 primary antibody (ab15580, 1:100 dilution; Abcam, Cambridge, MA, USA) overnight at 4°C. After washed with PBS, the sections were incubated with goat anti‐rabbit/FITC secondary antibody (111‐095‐003, 1:200 dilution; Jackson, West Grove, PA, USA) for 2 hrs at room temperature in darkness. The sections were then washed with PBS and incubated with EdU (Alexa Fluor^®^ 647, 1529380; Invitrogen) for 30 min. Finally, the sections were mounted with Prolong Gold containing DAPI (P36941; Invitrogen) and kept at 4°C in darkness. The confocal laser scanning microscope (LSM 710; Carl Zeiss, Jena, Germany) was used to observe the EdU‐positive cells and Ki‐67‐positive cells which determined cell proliferative rate in heart tissues.

### Wheat germ agglutinin (WGA) staining

The 5‐μm‐thick frozen heart sections were fixed with 4% PFA for 15 min. and then washed with PBS for three times. Next, the heart sections were incubated with WGA (L4895; Sigma‐Aldrich) solubilized in PBS for 20 min. at room temperature in darkness. After washed three times with PBS, the sections were mounted with Prolong Gold containing DAPI (P36941; Invitrogen) and kept at 4°C in darkness. The confocal laser scanning microscope (LSM 710; Carl Zeiss) was used to examine the cardiomyocyte size. A total of 30 fields per section were observed at 400× magnification and analysed with IMAGEJ software (National Institutes of Health). The cardiomyocyte size was determined by the measurement of total area divided by the number of cardiomyocytes.

### RNA extraction and qRT‐PCR

Total RNA were extracted from heart tissues using RNAiso Plus Total RNA extraction reagent (Takara, Kusatsu, Japan) according to the manufacturer's instruction. Then, 400 ng of total RNA was reverse‐transcribed into cDNA using Prime Script II First Strand cDNA Synthesis Kit (Takara). The quantitative PCR was then performed with PCR primers and SYBR Green (Takara) for 40 cycles in CFX96™ Real‐Time PCR Detection System (Bio‐Rad, Berkeley, CA, USA). The primer sequences used are as follows (5′–3′): ANP (forward: TGA CAG GAT TGG AGC CCA GAG and reverse: AGC TGC GTG ACA CAC CAC AAG) and BNP (forward: GCT GCT TTG GGC ACA AGA TAG and reverse: GGT CTT CCT ACAACA ACT TCA). The GAPDH (forward: 5′–3′ AGG TCG GTG TGA ACG GAT TTG and reverse: TGT AGA CCA TGT AGT TGA GGT CA) was used for normalization. The relative expression levels of ANP and BNP were calculated using the 2^−ΔΔCt^ method.

### Western blot analysis

Heart tissues were lysed in RIPA buffer with 1% PMSF and Pierce™ protease and phosphatase inhibitor (88668; Thermo, Waltham, MA, USA). The equal amount of total proteins was separated in 10% SDS‐PAGE gel and then transferred onto PVDF membrane. After blocked in 5% BSA for 2 hrs at room temperature, the membranes were blotted with mouse monoclonal anti‐CITED4 (ab77018, 1:1000 dilution; Abcam) and mouse monoclonal anti‐C/EBPβ (ab15050, 1:1000 dilution; Abcam) primary antibodies at 4°C overnight. The GAPDH (AP0063, 1:1000 dilution; Bioworld, St. Louis Park, MN, USA) was used as a loading control. The membranes were then washed with PBS and incubated with proper secondary antibodies for 2 hrs at room temperature. Then, the protein bands were visualized *via* enhanced chemiluminescence (ECL) kit in ChemiDoc XRS Plus luminescent image analyser (Bio‐Rad), and then quantified with IMAGE LAB software (Bio‐Rad).

### Statistical analysis

All data were analysed with SPSS software (Version 19.0) (International Business Machines Corporation, Armonk, NY, USA) and presented as mean ± S.E.M. The one‐way anova followed by Bonferroni's *post hoc* test was conducted to determine groups with significant difference. A *P*‐value less than 0.05 was considered statistically significant.

## Results

### Exercise‐induced cardiac growth is not influenced by inhibition of cell proliferation

**Figure 1:**
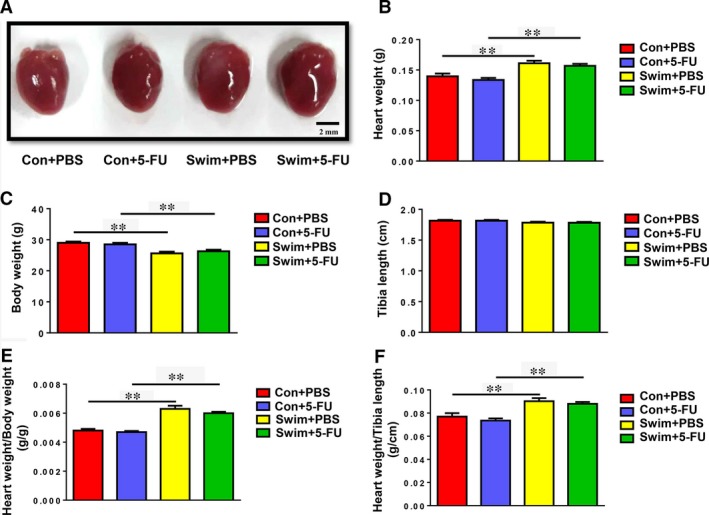
Effects of 5‐FU on exercise‐induced cardiac growth. The general size of heart (**A**), heart weight (**B**), body weight (**C**) and tibia length (**D**) were examined in sedentary control mice and swum mice with or without 5‐FU injections. The heart weight/body weight ratio (**E**) and heart weight/tibia length ratio (**F**) were calculated to evaluate cardiac growth in response to exercise with or without 5‐FU injections. *n* = 7 per group. **P < 0.01.

In this study, a 4‐week swimming exercise induced marked cardiac growth, as demonstrated by enlarged general heart size (Fig. 1A) and increased HW (Fig. 1B). Exercise also slightly reduced body weight (Fig. 1C), while tibia length remained unchanged (Fig. 1D). Indeed, an increase in the HW/BW ratio and HW/TL ratio was detected in exercised mice (Fig. 1E and F). To clarify whether cardiac cell proliferation is necessary for exercise‐induced cardiac growth, 5‐FU was intraperitoneally injected to mice which caused inhibition of cell proliferation. Importantly, exercise‐induced morphological cardiac growth, as well as increased HW/BW ratio and HW/TL ratio, was not influenced by treatment with 5‐FU (Fig. 1). These data indicate that exercise‐induced cardiac growth still develops when the proliferation of cardiac cells is inhibited by 5‐FU.

### Exercise‐induced cardiac cell proliferation is blunted by 5‐FU

**Figure 2:**
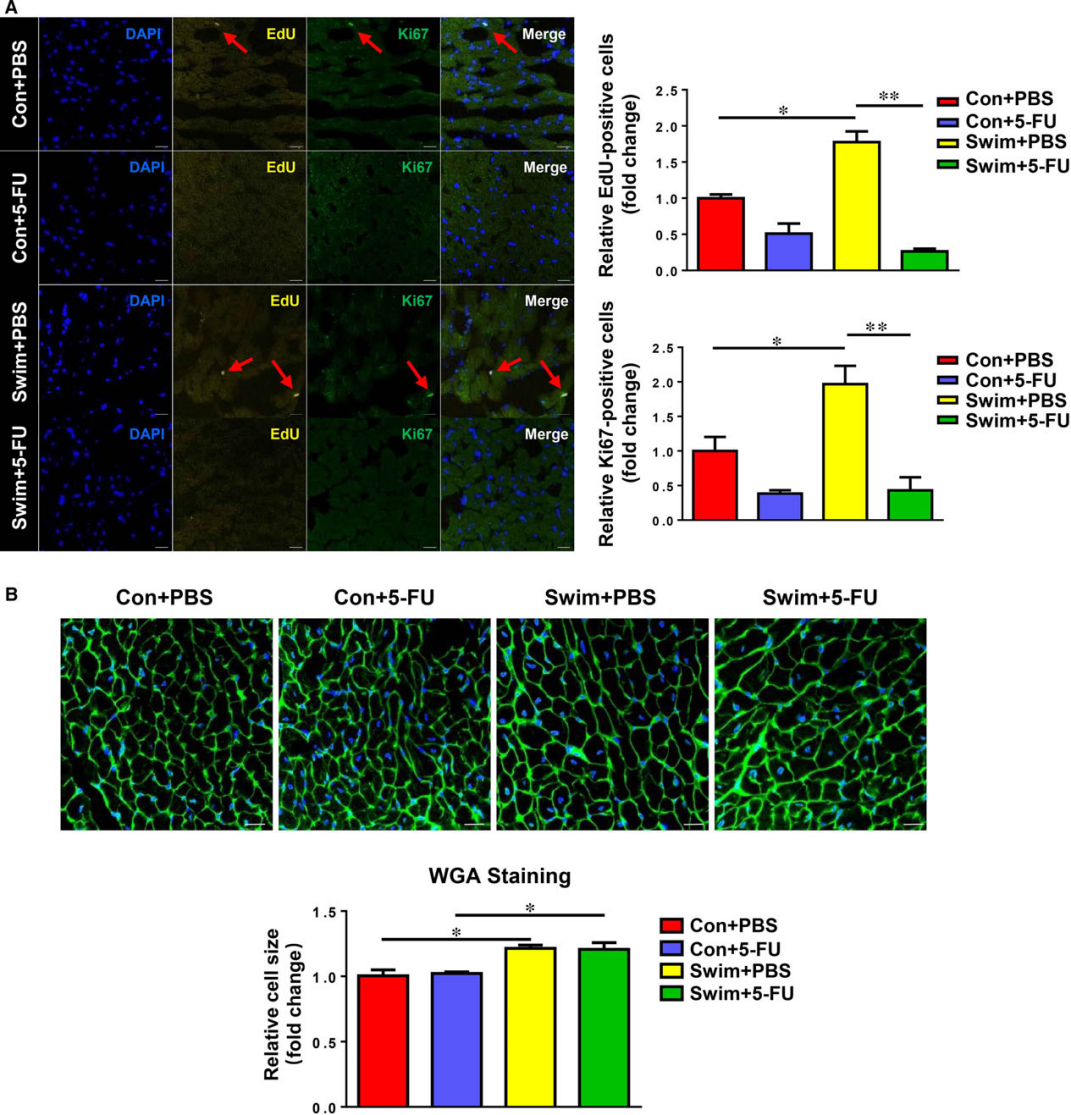
Effects of 5‐FU on cardiac cell proliferation and cell size. (**A**) The 5‐FU injections reduced EdU‐positive and Ki67‐positive cells in the exercised hearts. (**B**) The cardiomyocyte size was determined *via* wheat germ agglutinin (WGA) staining, showing that 5‐FU did not affect exercise‐induced enlargement in cardiomyocyte size. Scale bar = 20 μm. *n* = 3 per group. *P < 0.05; **P < 0.01.

Next, we examined both cardiomyocyte hypertrophy and proliferation upon exercise in the presence or absence of 5‐FU treatment. As demonstrated by EdU and Ki67 staining, a significant increase in EdU‐positive and Ki67‐positive cells was detected in the exercised heart, suggesting that exercise could promote cell proliferation in the heart (Fig. 2A). However, the increased cell proliferation in the exercised heart was significantly reduced by treatment with 5‐FU (Fig. 2A). On the contrary, exercise‐associated enlargement in cardiomyocyte size was not altered in 5‐FU‐treated mice as assessed by WGA staining (Fig. 2B).

### Cardiac cell proliferation is not necessary for exercise‐induced cardiac physiological growth

**Figure 3:**
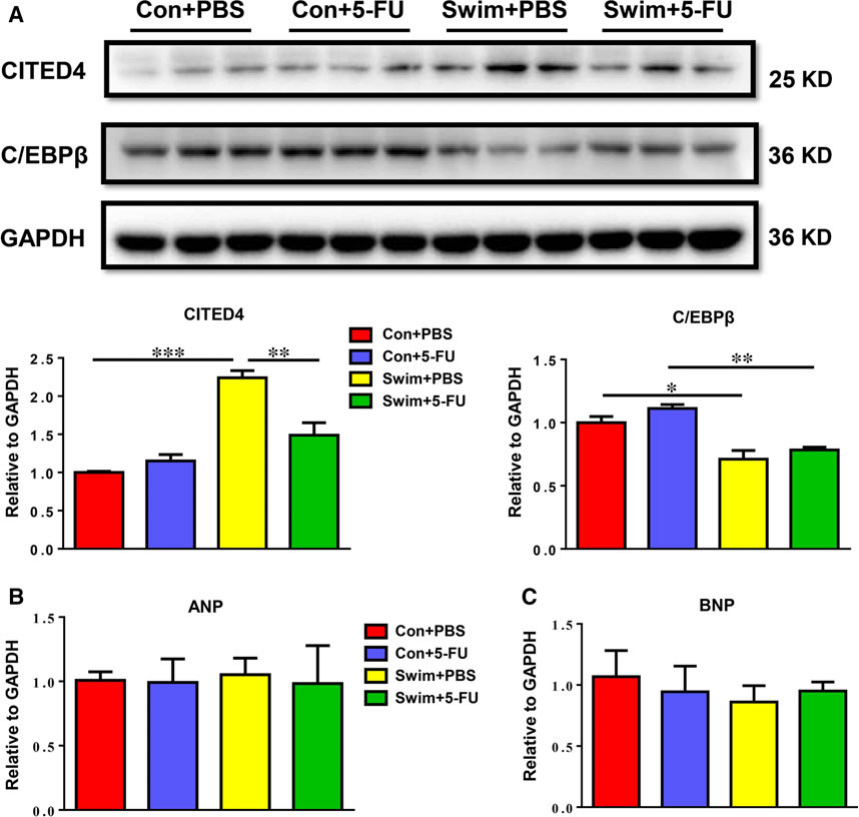
Cardiac cell proliferation is not necessary for exercise‐induced cardiac physiological hypertrophy. (**A**) The exercise‐associated regulation in CITED4, but not in C/EBPβ, was attenuated with 5‐FU injections as determined by Western blot (*n* = 3 per group). qRT‐PCRs demonstrated that the pathological hypertrophy markers ANP (**B**) and BNP (**C**) were not modulated with either swimming or 5‐FU injections (*n* = 5 per group). *P < 0.05; **P < 0.01; ***P < 0.001.

Besides direct detection of cardiac cell proliferation by EdU and Ki‐67 staining, the expression levels of C/EBPβ and CITED4 were also determined. The down‐regulated transcription factor C/EBPβ and subsequent activation of CITED4 have previously been reported as critical molecular mechanisms responsible for exercised‐induced cardiac growth 3. Consistently, here we found that C/EBPβ was down‐regulated, while CITED4 was up‐regulated in the exercised heart (Fig. 3A). Notably, the exercise‐associated activation of CITED4, a key regulator for cardiomyocyte proliferation, was blocked by 5‐FU (Fig. 3A). While exercise‐induced reduction of C/EBPβ, a transcription factor responsible for both cellular proliferation and hypertrophy, was not altered by treatment with 5‐FU (Fig. 3A). To determine whether exercise‐induced cardiac growth is still physiological when cardiac cell proliferation is inhibited, two typical markers for pathological hypertrophy including ANP and BNP were determined and both were not elevated in the exercised heart, regardless of 5‐FU treatment (Fig. 3B and C).

### Cardiac cell proliferation is required for the protective effect of exercise against cardiac I/R injury

**Figure 4:**
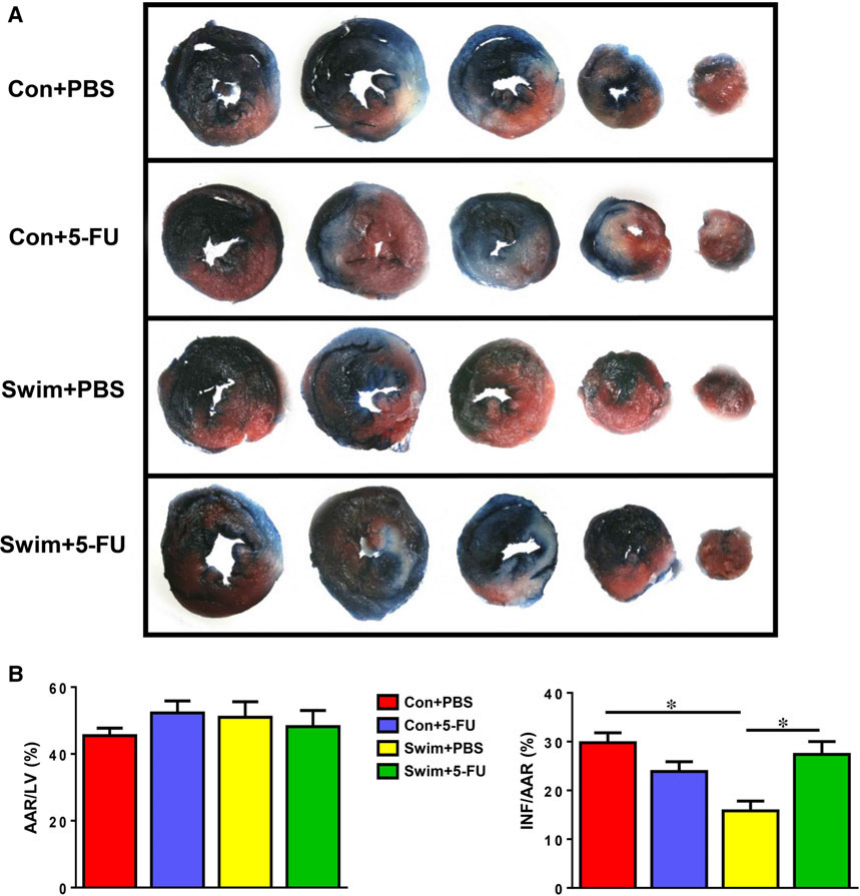
Cardiac cell proliferation is required for the protective effect of exercise against ischaemia/reperfusion (I/R) injury. (**A**) General aspect of I/R heart samples stained with 2,3,5‐triphenyltetrazolium chloride (TTC) from control sedentary mice and swum mice with or without 5‐FU injections. (**B**) The area at risk/left ventricle weight (AAR/LV) and the infarct size/area at risk (INF/AAR) ratios were calculated to determine the degree of cardiac I/R injury. *n* = 6–7 per group. *P < 0.05.

To further investigate whether exercise‐induced cardiac cell proliferation is essential for the cardioprotective effect of exercise, sedentary or exercised mice were submitted to cardiac I/R injury with or without 5‐FU treatment. Twenty‐four hours after I/R injury, mice hearts were submitted to TTC staining which demonstrated that the infarct size/area at risk (INF/AAR) ratio was significantly reduced in exercised mice, while the protective effect of exercise was totally abolished by treatment with 5‐FU (Fig. 4). Thus, we suggest that exercise‐induced cell proliferation in the heart, at least partly associated with newly formed cardiomyocytes, is an indispensable mechanism which mediates the beneficial effect of exercise upon myocardial injury.

## Discussion

The novel finding of our study is that reduced cardiac cell proliferation due to 5‐FU treatment does not affect exercise‐induced cardiac growth and the expression levels of ANP and BNP, indicating that increased cardiac cell proliferation is not necessary for exercise‐induced cardiac physiological growth. However, exercise‐associated cardiac cell proliferation is a requirement for the protective effect of exercise against cardiac I/R injury.

Exercise‐induced physiological cardiac growth is similar in general morphological level to pathological cardiac remodelling, with the increase in both heart size and HW 17, 18. However, cellular adaptions of cardiac growth in response to exercise are notably distinct from those in pathological conditions 19, 20. The adult mammalian heart retains a limited regeneration capacity, which can be enhanced by exercise training 21, 22, 23, 24. Although increasing evidence indicates that exercise‐induced cardiac growth is achieved by both cardiomyocyte hypertrophy and formation of new cardiomyocytes, little is known about to what extent these newly formed cardiomyocytes contribute to exercise‐induced cardiac growth. Here we used 5‐FU, a widely used antineoplastic agent, to inhibit exercise‐induced cardiac cell proliferation including the formation of new cardiomyocytes. It is also worth noting that the proliferation rate of cardiac cells, although not statistically significant, demonstrated a trend to be reduced by 5‐FU in sedentary mice. We suggested that this phenomenon might be linked to the very low proliferation rate of adult cardiac cells at baseline 13. Interestingly, here we found that the HW/BW and HW/TL ratios of swum mice were still increased in the presence of 5‐FU due to the fact that exercise‐associated increase in cardiomyocyte size was not affected, indicating that exercise‐induced cardiac growth still developed. Moreover, the fact that the expression levels of ANP and BNP were not changed by 5‐FU in swum mice further confirmed that the cardiac growth was physiological. Collectively, this study shows that cardiac cell proliferation including newly formed cardiomyocytes is not necessary for exercise‐induced cardiac physiological growth.

It was previously reported that reduced C/EBPβ and subsequent increased CITED4 contribute to exercise‐induced cardiac growth 3. Reduction of C/EBPβ *in vivo* and *in vitro* leads to both hypertrophy and proliferation of cardiomyocytes 3. C/EBPβ was also demonstrated to exert its antiproliferative effect *via* inhibition of CITED4 3. Interestingly, increased expression of CITED4 is able to activate cyclin D1, thus promoting cardiomyocyte proliferation 25. Here we showed that C/EBPβ was reduced while CITED4 was increased in swum mice heart, which is consistent with the previous report 3. Importantly, exercise‐associated molecule expression change of CITED4, but not of C/EBPβ, was blocked by 5‐FU. We suggested that this might be due to the fact that CITED4 is preferentially a key regulator for cardiomyocyte proliferation, while C/EBPβ is responsible for both hypertrophy and proliferation of cardiomyocytes 3.

The adult heart has an endogenous limited regeneration capacity in response to exercise, and exercise can protect myocardial injury 26, 27. Exercise was reported to reduce the apoptosis of cardiomyocytes, enhance mitochondrial biogenesis and improve myocardial energy metabolism, thus leading to cardioprotection against I/R injury 13, 28, 29. However, little is known about to what extent cardiac cell proliferation, including formation of new cardiomyocytes, contributes to the cardioprotective effect of exercise. Our data demonstrate that the benefit of exercise in reducing cardiac I/R injury could be abolished by 5‐FU treatment, highly suggesting that increased cardiac cell proliferation in response to exercise is essential to achieve the cardioprotective effect of exercise upon I/R injury.

One limitation of the present study is that the inhibitory effect of 5‐FU on cell proliferation is systemic and non‐cell type specific. This means that in addition to the inhibition of formation of new cardiomyocytes, the proliferation of other cell types could be reduced in the exercised heart with 5‐FU treatment. The cardiomyocyte renewal upon exercise may be linked to the proliferation of pre‐existing cardiomyocytes and the activation and differentiation of cardiac progenitor/stem cells 3, 23, 24, 30. Moreover, the bone marrow‐derived endothelial progenitor cells (EPCs) can also be recruited into systemic circulation in response to exercise, and mobilized to the heart where they contribute to endothelial renewal 31, 32, 33. Thus, the reduced cardiac cell proliferation in 5‐FU‐treated exercised heart as well as the loss of protective effect of exercise against I/R injury might also be attributable to the reduction of proliferation of other types of cells. Another limitation is that cardiac function was not directly assessed in this study. As 5‐FU has previously been associated with some degree of cardiac toxicity, the potential cardiotoxicity of this agent should be taken into account 34. However, we consider this unlikely in the current study for a number of reasons. First, it was previously demonstrated that a similar 5‐FU protocol was not associated with any cellular or functional cardiotoxic effects 16. Second, the frequency and duration of 5‐FU treatment in the present study were different from other studies reporting the cardiotoxicity of this agent 35, 36. Third, there was no elevation of ANP or BNP expression in control or swum mice receiving 5‐FU.

In conclusion, this study demonstrates that increased cardiac cell proliferation is not necessary for exercise‐induced cardiac physiological growth. However, cardiac cell proliferation is a requirement to achieve exercise‐associated cardioprotection against I/R injury.

## Conflict of interest

The authors declare there are no conflict of interests.
